# Hydrothermal growth of KTiOPO_4_ crystal for electro-optical application

**DOI:** 10.1038/s41377-022-01022-0

**Published:** 2023-01-12

**Authors:** Haitao Zhou, Xiaoling He, Wenyuan Wu, Jingfang Tong, Jinliang Wang, Yanbin Zuo, Yicheng Wu, Changlong Zhang, Zhanggui Hu

**Affiliations:** 1 Tianjin Key Laboratory of Functional Crystal Materials, Institute of Functional Crystals, School of Materials Science and Engineering, Tianjin University of Technology, 300384 Tianjin, China; 2 Guangxi Key Laboratory of Superhard Materials, China Nonferrous Metals (Guilin) Geology and Mining Co., Ltd, 541004 Guilin, China

**Keywords:** Nonlinear optics, Photonic crystals

## Abstract

“New” electro-optical (EO) crystals are hard to find, “old” EO crystals are scarce and each has its own problems, and the demand for high-performance EO crystals by higher power, higher repetition rate, and narrower pulse width laser is realistic and urgent. The EO performance of KTP was recognized as soon as it was discovered, but after more than 40 years of development, the reports, and products of EO devices based on KTP are less than those of other EO crystals, even though KTP is now almost the cheapest nonlinear optical crystal material. In this paper, based on our understanding of the crystal structure of predecessors and ourselves, especially the understanding and practice of quasi-one-dimensional ionic conduction mechanism, we think that crystal growth is the most important reason that affects the controllability of crystal performance. Through a series of science and technology, we realize the growth of large-size crystals with high-optical uniformity, then reduce the absorption of KTP to a very low level, and grow crystals with resistance to electric damage and laser damage. On this basis, reducing the conductivity and improving the uniformity of optical, electrical, piezoelectric, and ferroelectric properties are emphasized. The extinction ratio, piezoelectric ringing effect, and thermal influence of the EO switch based on KTP crystal are tested, and some publicly available progress of using KTP EO devices in high-repetition rate laser is listed. Finally, we are looking forward to the development of KTP EO crystal for the laser system to EO generator for integrated optics.

## Abstract (web summary)

Hydrothermal-grown KTiOPO_4_ (KTP) Crystal for Electro-optical Q-switch—a crystal having a high electro-optic coefficient, a low dielectric constant, and capable of operating at high frequencies (>1 MHz).

## Graphical abstract

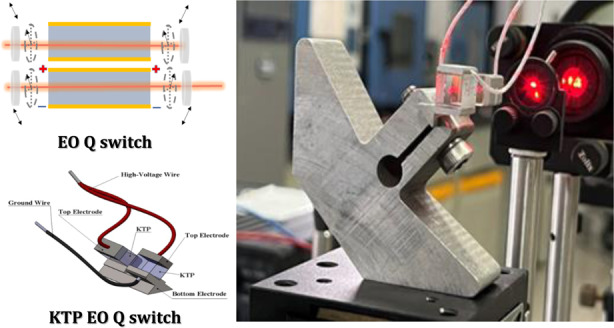

## Introduction

**Fig. 1:**
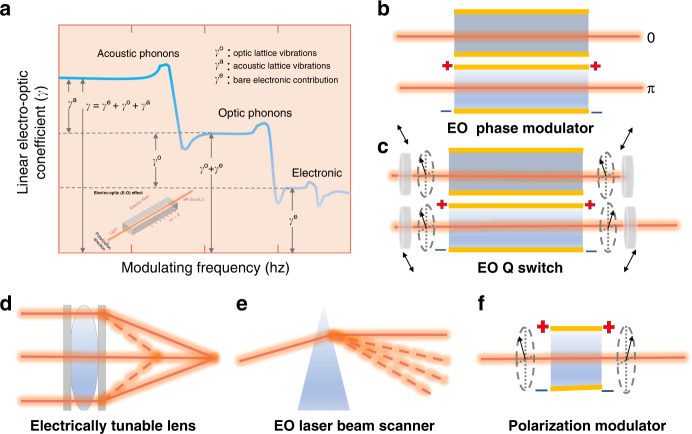
Electro-optic effect and some application. **a** Three contributions of Electro-optic (EO) coefficients: acoustic phonons, optic phonons, and electronic. **b**–**f** EO effect devices: **b** high-speed EO phase modulator, where an electric field changes the phase delay of a laser beam sent through the crystal; **c** EO Q-switch are modulators where the transmission is either switched on or off, rather than varied gradually. **d** Electrically tunable lenses, comparing to the traditional solid lenses with fixed refractive index, the refractive index of lenses is electrically tunable. Therefore, the focal length of lens is tunable by properly controlled the optical phase as aspheric or Fresnel lens phase profile. **e** EO laser beam scanner refract light by applying a phase delay by changing the refractive index of the medium, and by adjusting the voltage applied to the crystal to change the steering angle to EO control the phase delay across the beam cross-section. **f** Polarization modulator can thus be seen as a voltage-controlled waveplate, and it can be used for modulating the polarization state

Electro-optical (EO) crystals are a kind of crystal with EO effect, which are of essential importance for optoelectronic devices^1–4^. The EO effect encompasses several distinct phenomena, which can be subdivided into changes in absorption and changes in refractive index^5,6^. As far as the changes in refractive index are concerned, there are two main EO effects^6^, the quadratic EO effect (Kerr)^7–9^ and the linear EO effect (Pockels)^10–12^. When the linear EO effect is present in a solid, it usually dominates the quadratic EO effect, typically neglected. Within the Born–Oppenheimer approximation, the EO coefficients can be expressed as the sum of three contributions: acoustic phonons, optic phonons, and electronic see Fig. 1a^13,14^. Early attempts to use Pockels effect devices were not overly successful in the absence of a source of well-collimated, monochromatic light^15,16^. The advent of the laser opened the field for a variety of Pockels effect devices^17^. After that, EO effect devices promise broad applications in laser, optoelectronics, and optical communication, as shown in Fig. 1b–f. Two main material groups: semiconductors and dielectrics have been studied concerning their usefulness as EO active or passive components. The discussion is limited to bulk crystals of EO dielectric materials with Pockels effect useful for optoelectronics. The current EO dielectrics crystals were selected from the known nonlinear optical (NLO) crystals because they all belong to acentric point groups. Presently, the most commonly used EO crystals are KD_2_PO_4_ (DKDP)^18,19^, LiNbO_3_/LiTaO_3_ (LN/LT)^20–27^, β-BaB_2_O_4_ (BBO)^28,29^, KTiOPO_4_/RbTiOPO_4_ (KTP/RTP)^30–40^, and La_3_Ga_5_SiO_14_ (LGS)^41,42^, in addition to semiconductor EO crystals. The electro-optical properties of commonly used commercial EO crystals are shown in Table 1. Although there are commercial EO crystals available in the market, the developing requirement from current applications is still not satisfied^14,43,44^. Especially, the current laser system with high peak power and high-repetition rate puts forward new and higher requirements for EO crystals.

**Table 1:** Electro-optic performance comparison of KTP and other proven EO crystals^118^

		DKDP	LN	β-BBO	LGS	RTP	F-KTP	H-KTP
Transparency	nm	200–2150	400–5000	190–3500	242	350–4500	350–2500	350–2500
Damage threshold	MW/cm^2^	5000	280	50000	950	1000	600	1500
*r*	pm/V	*γ*_63_ = 26.4	*γ*_33_ = 30.9	*γ*_22_ = 2.2	*γ*_11_ = 2.3	*γ*_33_ = 39.6^119^	*γ*_33_ = 36.3	*γ*_33_ = 36.3
*n*		1.50	2.20	1.67	2.54	1.89	1.86	1.86
*n* ^3^γ	pm/V	89	329	10	37.7	267	234	234
Conductivity	1/Ω·cm	10^−12^	10^−14^	10^−9^ ~10^−15^	10^12^	10^−7^ ~10^−11^	10^−6^ ~10^−8^	10^−11^ ~10^−13^

Among the valuable EO crystals, KTP/RTP crystal stands out in its ability to achieve amplitude modulation at extremely high frequencies, or repetition rates, of up to 1 MHz^45–47^. KTP has been introduced as nonlinear optical material in 1976 by Zumsteg et al.^48^ and promises high nonlinear optical effective coefficients (15.4 pm V^−1^ at 1064 nm), high-optical damage threshold, wide acceptance angle, and thermally stable phase-matching properties. In the past 40 years, most efforts have been made to explore various frequency conversion applications, making KTP one of the most used nonlinear optical crystals, especially for ~1 μm laser^49–51^. However, sizeable linear EO coefficients and low dielectric constants^32^, which make KTP attractive for EO applications such as EO modulators and Pockels cell (PC), have not been well explored for device applications. A particularly important advantage of KTP crystals in practical electro-optical applications is the stable physical properties. Its dielectric constant and electro-optic coefficient are insensitive to frequency, which make it has good frequency stability. Its electro-optic coefficient is insensitivity to temperature, which led a well temperature stability. Furthermore its piezoelectric coupling effect is weak, which ensure the sensor stable in the equipment with more vibration.

Three problems hinder the EO application of KTP: the crystal size^52–57^, electro-chromic effect^58–60^, and optical inhomogeneity^36,61^. Large crystal size can be used in laser systems with large spot diameter, which is beneficial to reduce half-wave voltage. The resistance to electro-chromic effects can withstand higher laser energy density and harsher operating environment (high and low temperature, low pressure) and prolong service life. Better optical inhomogeneity can improve the extinction ratio of EO switch and beam quality of the laser. These are the needs in the applications of KTP. In addition, we found some differences with traditional knowledge in the actual research process. We have also observed that static strain depolarization is a problem in KTP, unlike in DKDP and *z*-axis LN.

## Fundamentals of potassium titanyl phosphate crystals

**Fig. 2:**
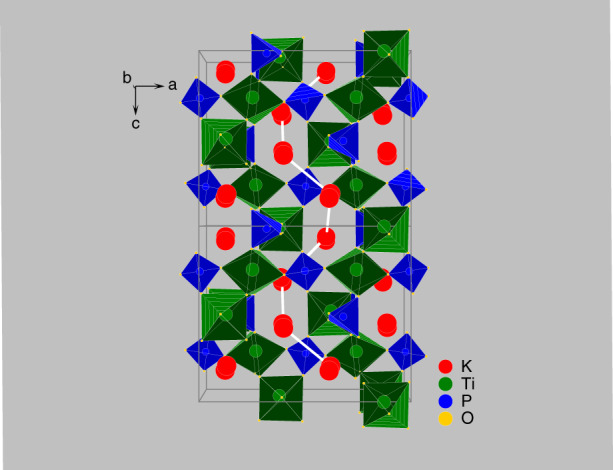
Structure and quasi-one-dimensional ionic conductivity of KTP. Structure of KTP, Ball-polyhedral representation of the KTP crystal structure with split K sites, showing continuous channels along the [001] directions

Structure–property relationships are the basis of understanding and solving problems of application KTP as EO materials. The performance potential of KTP, which belongs to the point group symmetry *mm2* and space group *Pna2*_*1*_, can be attributed to its extreme physical properties^62^. The KTP structural type normally covers compounds described by the general formula AMXTO_4_, where A is a single-charged metal cation or ammonium or a mixture of those, M is a double-, or higher-charged transition metal or non-metal cation combinations of and hose, X is an oxygen or fluorine atom, hydroxyl (OH) group or water (H_2_O), T is P, S, As, Si^63–65^. The compounds are formed by substituting the A position, which we call the isomorphs of KTP. Various properties of KTP and its important isomorphs (RTP, KTA, RTA, and CTA) are listed in Table 2. KTP structure is built of helical chains of the vertex-sharing TiO_6_ octahedra running along the *y*-axis corner-linked by the PO_4_ tetrahedra into a rigid three-dimensional (3D) framework hosting A^+^ ions in spacious cavities that merge into continuous channels along with all three main directions (Fig. 2). Moreover, the A^+^ ions can be distributed in media more complicatedly with several (up to 5) positions of various occupancies. At the same time, depending on the nature of A and M metals, the shape and size of these voids can slightly vary and several local energy minima can form accounting for the splitting of the A^+^ sites over two sub-sites symmetrically with equal occupancy (Fig. 2) or asymmetrically. We found that the KTP crystal will decompose after a period of the high electric field is applied. Suppose the K^+^ ions were substituted completely by other monovalent cations, the isomorphs. Fedotov et al.^65^ given the assumption that the A^+^ cation plays a crucial role in stabilizing this 3D structural framework.

**Table 2:** Crystal lattice parameters and selected physical properties of KTP and isomorphs, the most data are from the synthesis of literatures^118,119^

	KTP	RTP	KTA	RTA	CTA
Lattice constants					
*a*	12.819	12.952	13.125	13.264	13.486
*b*	6.399	6.4925	6.5716	6.682	6.682
*c*	10.584	10.555	10.786	10.7697	10.688
Curie temperature: °C^120^	955	785	873	750	637
IR cutoff edge: nm	4300	4300	5300	5300	5300
Birefringence *n*_z_ − *n*_x_ @1064 nm	0.0921	0.0884	0.0863	0.0782	0.0700
NLO susceptibilities @1064 nm:pm/V					
*d*_33_	16.9	17.1	16.2	15.8	18.1
*d*_32_	4.4	4.1	4.2	3.8	3.4
*d*_31_	2.5	3.3	2.8	2.3	2.1
*d*_eff_ for type-II SHG	3.35	2.51			
SHG cutoff along *x*-direction/*y*-direction: nm	1082/994	1147/1038	1134/1074	1243/1138	1548/1280
EO coefficients@633 nm:pm/V					
*γ*_33_	36.3	39.6	37.5	40.5	38.0
*γ*_23_	15.7	27.1	15.4	17.5	18.5
*γ*_13_	9.5	12.5	11.5	13.5	14.2
*γ*_42_	9.3				
*γ*_51_	7.3				

Furusawa et al. calculated the dimension of the bottlenecks of various hopping paths for the K ions. They found that one-dimensional (1D) spiral pathway channels with large bottlenecks could be formed along the *z*-axis^66^. However, practically ion migration does not necessarily follow all structurally “visible” pathways, and dynamics simulations are conducted to unveil the origin of the fast K^+^ diffusion kinetics by Zhang et al.^67^. Under static electric field, it was observed and confirmed by in situ X-ray diffraction^68,69^ and Raman spectra^70^ that the polarization of space charge was due to the movement of K^+^ ions in the 1D channel. These channels account for the high and anisotropic ionic mobility along that direction, with the *z*-axis conductivity *σ*_33_ being about three orders of magnitude higher than *x*-axis conductivity *σ*_11_ and *y*-axis conductivity *σ*_22_^32^, and KTP is described as a quasi-1D superionic conductor^66^.

## Crystal growth

KTP decomposes on melting at ~1150–1170 °C^71^, which inhibits the use of convenient melt-growth techniques, such as the Czochralski method, for the growth of KTP. Instead, crystals from the KTP family are grown by solution-growth techniques. As KTP hardly dissolves at ≤300 °C, the conventional solution method cannot be used either, and two different methods are commonly used, hydrothermal growth and flux growth. For ordinary nonlinear optical frequency conversion with low energy density, the crystal grown by flux method can meet the requirements because this application can tolerate a significant absorption coefficient at 532 nm without causing “gray trace” or other laser damage. The equipment required by this method is relatively simple, the cost is low, and the KTP molten salt growth process is mature, which can meet the application requirements of low-cost, batch, and high-consistency KTP frequency conversion devices. The KTP crystal grown by the hydrothermal method can meet the laser frequency conversion requirements of higher energy density than the molten salt method KTP. At the same time has a lower resistivity, which is beneficial to electro-optical applications.

### Flux crystal growth for reducing ionic conductivity

While the adoption of the flux method for KTP growing has played a key role in the practical application of nonlinear optical crystals, especially the popularity of green solid-state lasers in consumption and processes. In addition, LiB_3_O_5_ (LBO)^72^ and β-BaB_2_O_4_ (BBO)^73^ are commonly used nonlinear optical crystals grown by flux method. The flux method has played an irreplaceable role in the material preparation of nonlinear optical crystals. At the same time, these three nonlinear optical crystals, in turn, the flux method, retains their status as an essential method for industrial crystal growth. The significant advantage of the flux method is that crystal growth can be achieved under ambient pressure. Nine years after the hydrothermal growth of Rb_1−x_K_x_TiOPO_4_ (1.0 ≥ *x* ≥ 0), RKTP crystal was first reported, and flux method have been employed by groups in both the Soviet Union^74^ and China^75,76^. Crystal size is no longer the obstacle to EO devices fabricated by F-KTP^71^. After the flux method grows crystals of sufficient size, the EO properties and EO applications of F-KTP have also been studied. When operated in a thermally compensated mode, good Q-switching properties have been demonstrated with KTP crystals in a thermally compensated mode^30^. Salvestrini et al. take a comparative study of five nonlinear optical crystals for EO Q-switching of laser resonators. KTP exhibits the highest values for the EO application’s merit figures, including driving voltage, switching speed, and piezoelectric ringing (PER)^77^. Unfortunately, due to the relatively high conductivity, when F-KTP was used as a high average power EO switch, electrons injected at the anode of the crystal can reduce the stoichiometric Ti^**4**+^ ions in the crystal lattice to Ti^**3**+^, even if F-KTP grown at temperature constant is used instead of traditional the temperature drop flux method^78^. Upon applying an electric field, a black absorbing band moves from the anode towards the cathode in the F-KTP, compromising its utility as a Q-switch. This phenomenon is the electro-chromic damage mentioned above.

### Basic aspect of hydrothermal growth for large KTP Crystal

Hydrothermal growth (or Solvothermal, if a solvent other than water is used) is commonly used to grow high-temperature crystalline materials, especially commercial quantities of pure quartz. The advantages of the hydrothermal technique are a “relatively low” growth temperature, a Δ*T* close to 0 at the solid/liquid interface, an “easily scalable” approach, and the potential to reduce the source of most of the impurities. Thus, electro-chromic damage cannot be observed, mainly because the *σ*_*33*_ of H-KTP is 3–5 orders of magnitude lower than F-KTP. Therefore, the key challenge lies in developing H-KTP that can obtain sufficient size and homogeneity crystals of KTP with a big Z-cut cross-section.

**Fig. 3:**
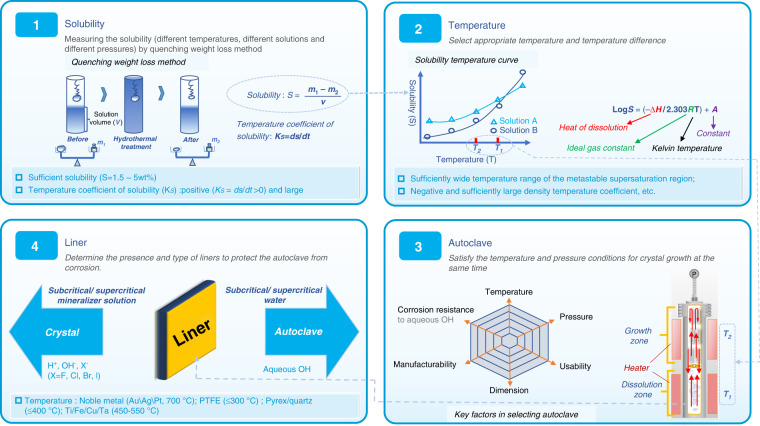
Pre-work of hydrothermal bulk crystal growth. Before growing crystals by hydrothermal method, the following four tasks should be done first: measuring the solubility (different temperatures, different solutions, and different pressures); select appropriate temperature and temperature difference; select an autoclave to meet the crystal growth requirements; determine the presence and type of liner according to the situation

**Fig. 4:**
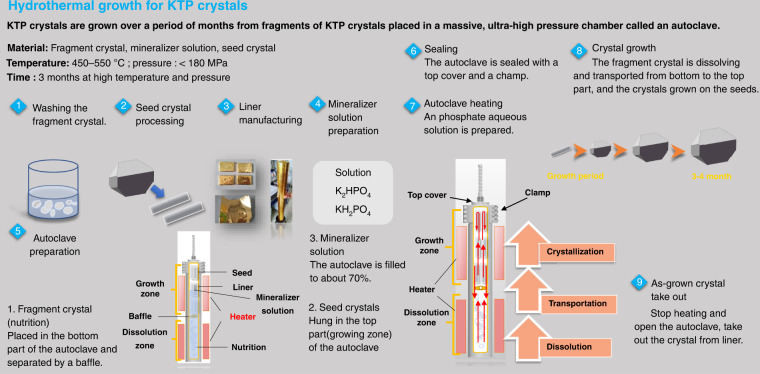
Production process of bulk KTiOPO4 crystal by hydrothermal method. Hydrothermal method is the process of crystal growth under hydrothermal conditions occurs at high regimes and pressures. Therefore, this process leads in special thick-walled vessels – autoclaves, made of special high-strength corrosion and heat-resistant steel. The typical hydrothermal growth process of KTP crystal includes nine steps, starting with the preparation work of nutrients, seed, liner, mineralizers, and autoclave etc. (step 1–5), and finally taking out the grown crystals (step 9) after autoclave sealing, heating and crystal growth (step 6–8)

**Fig. 5:**
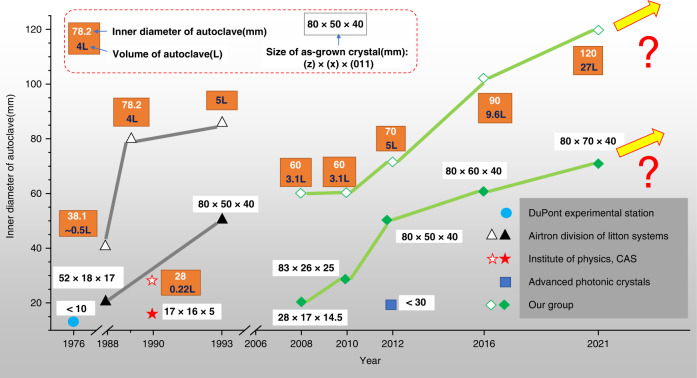
Scale-up of autoclave and as-grown H-KTP in the past 46 years. The horizontal axis is the year, and the vertical axis is the inner diameter of the autoclave. The size of the crystal is largely determined by the inner diameter of the autoclave, so it is listed together in the graph, marked with a solid symbol to distinguish it from the hollow symbol representing the inner diameter of the autoclave. Due to the difficulty of equipment and technology, there are only a handful of institutional teams involved in hydrothermal growth of KTP after past 46 years

**Fig. 6:**
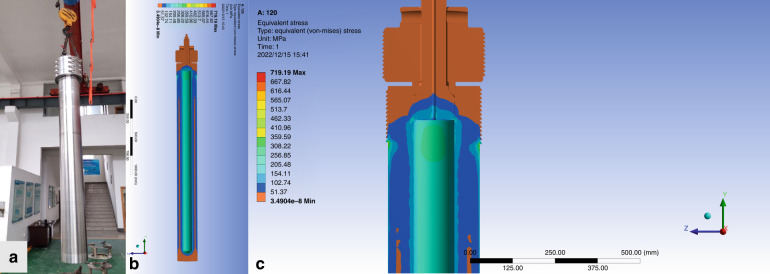
The stress strain simulation analysis of large autoclave for large KTP crystal growth. **a** Autoclave with effective internal dimensions of Φ120 mm diameter × 2.4 m high. Its safe long-term service temperature is less than 650 °C and its pressure is less than 300 MPa. Simulation of the autoclave (**b**) and the seal part (**c**) is completely under compression stress

**Fig. 7:**
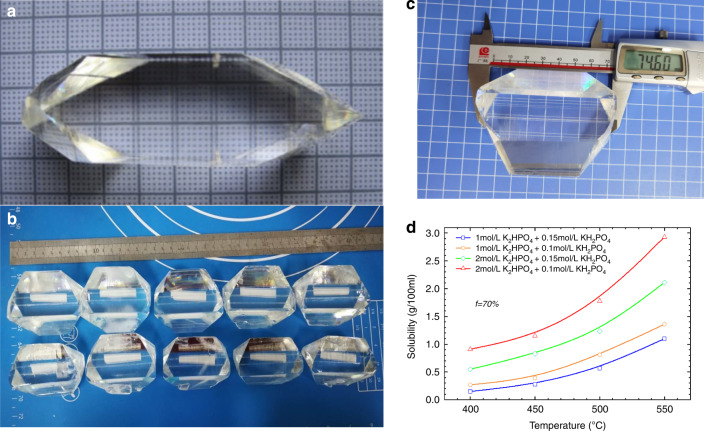
As-grown KTP crystal by hydrothermal method and solubility-temperature curve. As-grown KTP crystal in one autoclave **a** with Φ 60 mm inner diameter; **b**, **c** with Φ 90 mm inner diameter in a cycle of growth. **d** Solubility-temperature curve for KTP in K_2_HPO_4_ and KH_2_PO_4_ mixed solution in the range from 400 to 550 °C

For hydrothermal crystal growing, temperatures of 300–700 °C are required, which generates high pressures of 30–300 MPa that the autoclave must contain. Its ability to create supercritical water from high pressures and temperatures due to the decrease in water viscosity allows for insoluble materials at supercritical temperature and pressure to become soluble in solution. Mineralizer solutions such as fluorides, chlorides, hydroxides, and carbonates, are still needed to help increase the solubility of the starting material in cases where the low dielectric constant of water causes a low solubility of compounds even at supercritical temperatures. It is also necessary to use some capsule or liner made of a non-reactive precious metal (e.g., titanium, silver, gold, or platinum) to protect the autoclave against corrosion by supercritical mineralizer solutions. For the growth of bulk single crystals, the temperature gradient causes the convective flow of the saturated solution upwards into the colder zone is a pivotal step, where it is super-saturated, and the material will attempt to precipitate. Seed crystal provides a site for precipitation, which causes it to grow in the designed direction rather than spontaneously nucleating for haphazard production. As shown in Fig. 3, temperature and pressure, autoclave, capsule or liner, mineralizer, and seed constitute the fundamental aspects of hydrothermal crystal growth. The most apparent reason hydrothermal crystal growth is not as extensively studied as the melt techniques are that it involves a highly specialized set of equipment and knowledge, requiring an extensive initial financial investment to obtain the necessary high-pressure equipment and time investment to learn about its safe operation. The hydrothermal KTP crystal growth production process is shown in Fig. 4, respectively. The scaling of autoclave and as-grown H-KTP in the past 46 years is illustrated in Fig. 5, and the biggest autoclave for H-KTP, which practical internal dimensions of Φ120 mm diameter × 2.4 m high, is shown in Fig. 6. In 2008, an H-KTP crystal with 83 × 26 × 25 mm^3^ in dimension and 132 g in weight was obtained from an autoclave with an inner diameter of 60 mm and a volume of 2.4 L after 90 days of growth^55^, shown in Fig. 7a. The typical size of the crystal grown is 70 × 70 × 40 mm^3^ (*x, z, y*), and Fig. 7b, c shows ten crystals produced at one turn in an autoclave with an inner diameter of 90 mm a volume of 5 L. Now for 120 mm × 2.4 m autoclave, crystals of the above size can be grown at one time, reaching 32–40 pieces, with a total weight of about 10 kg.

The relationship between the solubility of the crystallizing substance and the temperature is the most crucial characteristic of a hydrothermal crystal growth system. The main objective of the solubility study dependence on pressure and temperature was to find a suitable mineralizer solution to increase the solubility, growth rate, and crystal quality but at reduced pressure and temperature conditions. Prior to this, only Gier^79^ used a high-concentration composite mineralizer of 7.14 M K_2_HPO_4_ + 7.14 M KH_2_PO_4_ + 1.15 M K_6_P_4_O_13_ in the patent to make a growth attempt under the conditions of a dissolution zone temperature of 630 °C and a temperature difference of 80 °C and obtained the size <5 mm H-KTP crystal. However, there is no relevant solubility in the K_2_HPO_4_ + KH_2_PO_4_ mixed solution, then we measured the solubility of KTP crystals, and the results are shown in Fig. 7d. Under the temperature *T* = 450–550 °C, when the concentrations of K_2_HPO_4_ and KH_2_PO_4_ are 2 mol·L^−1^ and 0.1 mol·L^−1^, respectively. The solubility value of KTP crystal changes in the range of *S* = 1.15–2.93 g/100 mL, and the solubility-temperature coefficient is also relatively large (>0.15 g/100 mL/10 °C). The addition of 0.1 mol·L^−1^ KH_2_PO_4_ can inhibit the formation of impurity K_2_Ti_6_O_13_ and ensure the considerable solubility of KTP in this system^80^. Moreover, to adjust the system’s redox potential, the oxidant H_2_O_2_ was added to reduce the probability of V_O_^2+^, which is believed to be the main reason for “Gray-track” damage and electro-chromic effect damage^80^. Frequently, favorable dissolution and growth zone temperatures for scale-up transport reactions are gleaned from the solubility study. More importantly, unfavorable temperature zones are discarded from the reaction scheme to conserve time and resources.

### Rb-doped KTP grown by micro-doped sustained-release hydrothermal technology for EO application

There are two ways to achieve the goal of reducing *σ*_33_. The first is to reduce thermal defects, non-stoichiometric defects, and impurity defects by controlling the growth conditions and growing “perfect” crystals. Another way to accomplish low *σ*_33_ is to reduce the “bottleneck” size and increase the ion size in the channel by doping other favorable atoms. By understanding the structure, there are two main mechanisms for these doping to change the *σ*_33_: one is to change the ion mobility in the “channel,” and the other is to change the “bottleneck” of the channel. Of course, it also includes the combination and interaction of the two mechanisms. For example, the *σ*_33_ of KTP grown by hydrothermal method (H-KTP) with low growth temperature (≤600 °C) is 3–6 orders of magnitude lower than that developed by flux method (F-KTP) with high temperature (>900 °C), which is mainly due to the decrease of K vacancy (V_O_^•^) concentration. Meanwhile, for the doping method, Ba, Fe, Cr, and V are considered unfavorable to the reduction of *σ*_33_, whereas Rb, Ga, Ce, etc., are favorable^49,60^. However, new problems will appear when doping is used, mainly the change of impurity in the crystal growth process and the resulting uneven distribution in the crystal. Therefore, whether it reduces the *σ*_33_ by growing perfect crystals or doping modified crystals, crystal growth always becomes the most crucial problem.

The *σ*_33_ of the hydrothermal method was considered to meet the application requirements of EO devices. However, in actual research, we found that although there was no electro-chromic phenomenon like F-KTP, it was observed that the ever-increasing turn-off voltage was needed to realize the turn-off of EO switches. We have grown crystals such as Ce, Sc, Nb, Na, Cs, and Rb and found that even when the doping concentration of Ce, Sc, Nb, and doping is deficient (≤0.1%), the *σ*_33_ of the crystals will not decrease significantly and crack. Still, when the doping concentration of Cs^+^ or Na^+^ is 0.04 M, the *σ*_33_ will not reduce significantly. In comparison, if the Rb^+^ ions concentration in the mineralizer solution is in the range of 0.01 to 0.1 mol·L^−1^, the *σ*_33_ can be reduced by 1–2 orders of magnitude compared with pure hydrothermal KTP, and no obvious cracks have been observed.

Depending on the distribution coefficient between solution and crystal, impurity/dopant depletion or enrichment may occur. This presents temporal inhomogeneity of dopant concentration to the growing crystal, resulting in spatial inhomogeneity of crystal properties. Therefore, to obtain crystals with high uniformity, an improved thermo-hydrothermal growth technique was adopted. Specifically, the dopant is not directly mixed into the solution or now put into the answer, but is put into a small capsule or other small containers. One or more openings of a specific diameter are reserved to slow down the dopant. It communicates with the substances in the whole system to realize the slow release of trace amounts of doping. We refer to this technology and a series of supporting technologies, such as the micro-doping sustained-release hydrothermal technology (MSHT). After adopting this technology, the *σ*_33_ uniformity of the crystal is improved, thus significantly increasing the success rate of EO crystal pairing into EO Q-switches from 50% to about 75%.

## States of the art of potassium titanyl phosphate crystals

### Optical properties

**Fig. 8:**
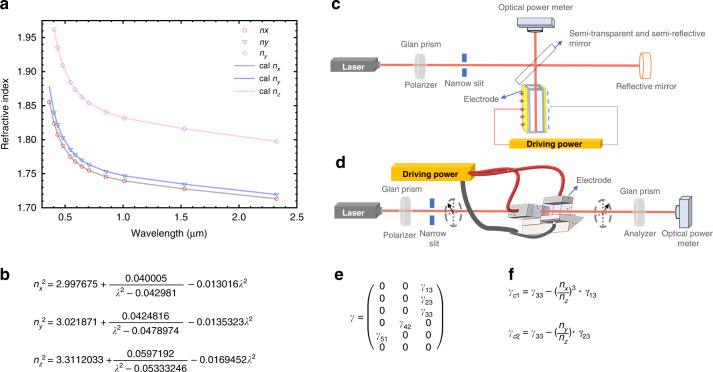
Refractive index and electro-optic coefficient measurements in H-KTP. **a** Refractive index versus wavelength and **b** Sellmeier equation of KTP; Schematic of our custom-built single beam amplitude modulator for measuring **c** EO coefficients *γ*_13_, *γ*_23_, *γ*_33_ and **d** effective EO coefficients *γ*_c1_ and *γ*_c2_; **e** EO coefficient matrix for KTP; **f** Calculation formula of the effective EO coefficients *γ*_c1_ and *γ*_c2_ through EO coefficients *γ*_13_, *γ*_23_, *γ*_33_

We have reported the transmittance from ultraviolet to infrared in the literature^55^. The transparency curve of the H-KTP crystal was very flat between 450 and 2500 nm wavelength without any valleys; a narrow valley near 2750 nm wavelength caused by OH^−^ indicated the H-KTP. It should be noted that, although a quantitative comparative measurement was not made, typical of H-KTP, the OH^−^ concentration was much more than that in F-KTP. Setzler et al. indicated that OH^−^ molecular ions in the hydrothermally grown crystals could charge-compensated V_K_^−^ and form Ti^3+^ center^81^. The thermal stability of the Ti^3+^ center in H-KTP is considerably less stable than that in F-KTP, which helps explain why gray tracks form in some F-KTP and not in H-KTP. Figure 8a shows the principal refractive indices of the H-KTP. Though Sellmeier’s equations (Fig. 8b) calculate its class II phase-matching angle of 23.3925° for the 532 nm laser generated by frequency doubling at 1064 nm at room temperature, which is smaller than the 26° reported by the earlier hydrothermal method, and also it is inconsistent with the results we obtained in the year of 2008, the reason for which is not apparent to us, but also suggests that the crystal structure and properties will change due to the difference of method conditions.

### EO properties

The E-O coefficient of KTP crystals was adopted to measure by the traditional half-wave voltage method, and the EO coefficient matrix is shown in Fig. 8e. We built two sets of the experimental device and optical path for measuring EO coefficients *γ*_13,_ *γ*_23_*, γ*_33,_ and effective EO coefficients *γ*_c1_ and *γ*_c2_ (Fig. 8f), respectively, displayed in Fig. 8c and Fig. 8d, after that, the effective EO coefficients were verified and compared with each other based on the individual test results combined with the derived effective EO coefficients equations. When the KTP crystal is used as an EO modulator, the applied electric field is parallel to the *z*-axis, and the light propagation direction is parallel to the *y*-axis or *x*-axis. If a relatively lower turn-off voltage is desired, a *y*-axis switch is required. In practice, long *x*-axis switches are often used to obtain the desired low turn-off voltage for crystal growth economics. The E-O coefficient of the H-KTP crystal can be determined as follow in Table 3. Our EO coefficient measurement results at 632.8 nm are consistent with the 1986 test results using the hydrothermal method KTP^32^.

**Table 3:** Electro-optic coefficient of H-KTP Unite: pm/V

EO coefficient	Our H-KTP	Airtron’s H-KTP^32^
*γ*_13_	11.7	9.5
*γ*_23_	16.23	15.7
*γ*_33_	37.815	36.3
*γ*_c1_	27.185	27.2
*γ*_c2_	22.968	21.1

### Damage resistance of hydrothermally grown KTP

Although the linear, nonlinear, and EO properties of KTP crystals grown by the flux and hydrothermal techniques are similar, differences have been observed in some dielectric properties and the very high-power optical damage characteristics. It is generally believed that the physical properties of H-KTP suffer as a byproduct of its growth process.

**Fig. 9:**
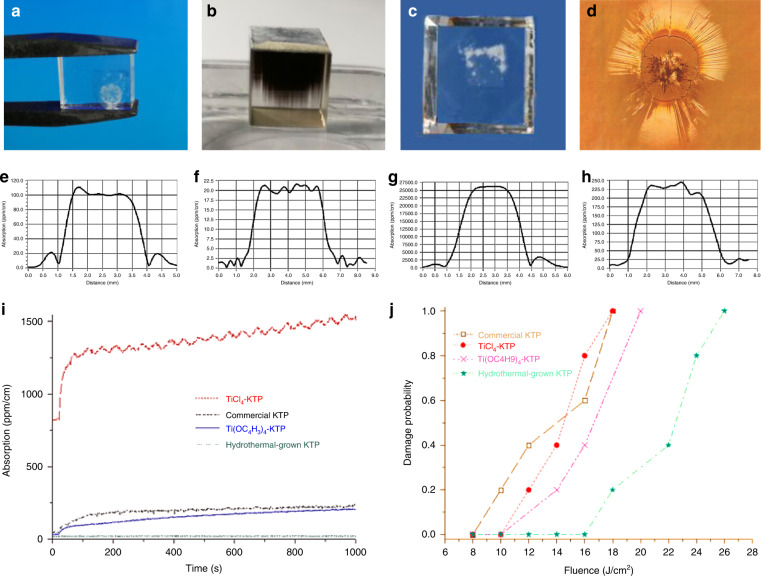
Damage resistance of KTP. Photograph of F-KTP under the 300 V·mm^–1^ electric field **a** after 10 min, **b** after 30 min, and **c** H-KTP. under the 1000 V·mm^–1^ after 72 h. **d** Microscope image of the catastrophic damage by laser. Bulk weak absorption values of **e** F-KTP and **f** H-KTP crystals at 1064 nm, **g** F-KTP, and **h** H-KTP crystals at 532 nm^95^; **i** Relaxation time of green induced infrared absorption: F-KTP (red line) and H-KTP crystals (green line)^95^; **j** Surface LIDT of the KTP samples at 1064 nm: F-KTP (black square) and H-KTP crystals (green pentagram)^95^. Fig **e**–**j**, reprinted with permission from ref. ^95^, © 2012 Society of Photo‑Optical Instrumentation Engineers (SPIE)The Optical Society Copyright
  Before the above test, we measured the dc conductivity of the three crystals at room temperature (RT) and found that the *σ*_33_ of H-KTP is about 10^−10^ S·cm^−1^, four orders of magnitude higher than that of F-KTP. In the early research, the *σ*_33_ of H-KTP grown by Airtron is only ~2.5 × 10^−9^ S·cm^−1^ at room temperature reported in 1986. They also said the *σ*_33_ is between ~10^−6^ and 10^−8^ S·cm^−1^ when the crystal is grown in the K_6_P_4_O_13_(12 mol·L^−1^ PO_4_^3+^) + 0.025 mol·L^−1^ KNO_3_ solution at the temperature of 580 °C. Meanwhile, *σ*_33_ of another crystal, grown from 2 mol·L^−1^ K_2_HPO_4_ + 0.125 mol·L^−1^ KNO_3_ mixed solution, is about 10^−11^ S·cm^−1^, 3–5 orders of magnitude greater than the crystals mentioned above. In 1996, the *σ*_33_ of H-KTP from Airton ranged from ~10^−10^–10^−12^ S·cm^−1^, similar to our data obtained from many tests.
- **Laser-induced photo-chromic damage at high peak power: “Gray-track” damage** Gray tracking should be called more precisely photo-chromic damage and describes the light-induced creation of relatively stable color centers that appear as gray lines in the laser light path. Due to the ease of color center generation by short wavelengths and the large absorption spectrum of the color centers, which covers part of the near-infrared wavelengths, the same physics is also discussed under the naming “green induced infrared absorption” (GRIIRA). The GRIIRA is an effect known to vary between samples. It is susceptible to the crystalline quality and stoichiometry of KTP as V_K_^−^ and V_O_^2+^ act as stabilizing defects facilitating the capture of free carriers, thus creating color centers. The weak absorption values of the KTP crystals were measured at 1064 nm and 532 nm by the photothermal common-path interferometer. As shown in Fig. 9i, only the H-KTP presented by a cyan line (the lowest line) behaves with efficiently high resistance of the gray-tracking^95^. Consequently, it can be concluded that all samples produced a gray-tracking effect except the H-KTP. In addition, the weak absorption value of the H-KTP was at least two orders of magnitude lower than that of F-KTP at 532 nm (Fig. 9g, h), while it was 1/5 of the F-KTP at 1064 nm (Fig. 9e, f)^95^.
  For commonly paired KTP EO devices, due to the sizeable thermo-optic coefficient of the KTP crystal, and the different energy of the oscillating laser and pumping light absorbed by the front and back crystals, the thermal depolarization effect will occur under the condition of high pumping intensity, which will lead to the deterioration of dynamic extinction ratio, the increase of insertion loss and the decrease of switching performance.
- **Catastrophic laser-induced damage at high peak power** Unlike “Gray-track” damage, catastrophic laser-induced damage (Fig. 9d) is characterized by plasma formation that usually leads to the creation of microcracks, or even completely broken. In the case of initial nonlinear application and EO applications of the KTP, the higher catastrophic laser-induced damage threshold (LIDT) is one of the advantages of KTP compared with low damage threshold crystals such as LN. The damage threshold of H-KTP is higher than that of F-KTP, attributed to the low conductivity. However, the more accurate explanation should be the low point defect concentration. Figure 9j shows the surface LIDT of four samples. The results illustrate that the H-KTP has the highest LIDT among them, two times higher than the commercial F-KTP. More details can be found in ref. ^95^.

- **Ionic conductivity and electro-chromic damage at high average power and high-repetition rate**. Ionic conductivity and electro-chromic effect damage at high average power, high-repetition rate: Because of the largest EO coefficient *r*_33_, the optimum configuration is propagation direction *y-*axis or *x-*axis, applied electrical field and polarization of the light field *z-*axis. Hence, low conductivity σ_33_ can increase the threshold of electrical damage and reduce the possibility of electrical damage. Unfortunately, *σ*_*33*_ is higher than σ_11_ and *σ*_22_, and it also depends on the growth conditions and methods^60^. So, changing the conditions of growth is the first way to avoiding electro-chromic damage, and the scheme of reducing conductivity by doping is mainly explained. In this crystal, the K cation may be partially or fully replaced by Rb, Na, Tl, Cs, Ag, and NH_4_ cations^63,82^, the P atom may be substituted by As atom^83^. Finally, the Nb^84^, Zr^85^, Sb^86^, Yb^87^, Al^88^, Cr^89^, Ta^86^, Ge^90^, Sn^91^, Ga^88,92^, Sc^89^, Ce^92^, Te^93^, and Fe^94^ cations may substitute the Ti atom of KTP. Furthermore, another path that is lessused is hydrothermal method, instead of the flux method for crystal growth.
  Figure 9 shows the electro-chromic damage present in F-KTP and H-KTP. Two F-KTP crystals we obtained were placed under an applied field of 300 V·mm^−1^, one showed a white substance on the surface after 10 min of stopping pressing (Fig. 9a), and the other was entirely blackened after 30 min (Fig. 9b). In contrast, no change was observed for H-KTP at the same electric field strength after 72 h. Then the electric field strength was increased to 1000 V·mm^−1^, more than three times the original. Finally, after 72 h, a phenomenon similar to F-KTP under 10 min and 300 V·mm^−1^ was observed in Fig. 9c. Lastly, it is widely accepted that F-KTP possesses a lower damage threshold than H-KTP.

### Optical homogeneity

**Fig. 10:**
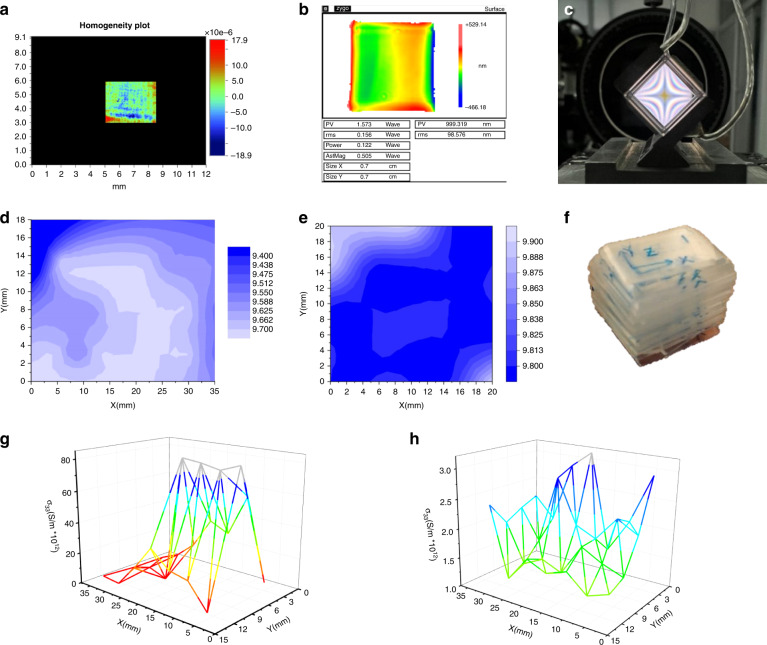
Homogeneity of H-KTP. Optical Homogeneity of H-KTP (**a**) and H-RKTP (**b**); **c** Conoscopic interference image of a H-RKTP EO switch. Piezoelectric coefficient d_33_ distribution of **d** H-KTP and **e** H-RKTP; **f** 20 wafers with a thickness of 1 mm along the *z*-plane from beginning to end for confirming the ferroelectric domain of the crystal by measuring the piezoelectric coefficient *d*_33_; Distribution of DC conductivity at 70 °C of H-RKTP crystal **g** by traditional hydrothermal method and **h** micro-doping sustained-release hydrothermal technology (MSHT)

Here, the optical uniformity discussed is primarily the uniformity of the refractive index, as this is the most relevant for EO performance. Since hydrothermal crystallization is carried out at relatively low temperatures, much below the melting point, and kept almost constant (±0.2 °C) for several months, the crystals show no intense thermal stresses, plastic deformations, or structural defects of various kinds (blocks, waviness, etc.) unless defects in the seed crystal cause them. Figure 10a shows the homogeneity plot of the H-KTP crystal, the optical homogeneity characterized by the root-mean-squared of the gradient of refractive index was about 4.15 × 10^−6^ cm^−1^. Figure 10b shows the uniformity of H-RKTP is 9.1 × 10^−5^ cm^−1^. The result indicated that the optical homogeneity of this KTP and H-RKTP crystal was perfect in the whole area investigated when the crystal is made into the electro-optical device, observing the conoscopic interference fringes against the background of a white light source, how the fringes are almost free of eccentricity, which indicates that it has good optical homogeneity, which is important for obtaining an EO switch with a high extinction ratio.

### Piezoelectrical and ferroelectric homogeneity

Also, the Currie temperature Tc of KTP is higher than the reaction temperature for hydrothermal growth. On the contrary, the growth temperature of the flux method generally needs to exceed Tc, KTP loses the uniformity of its ferroelectric domains, and electron dipole movement becomes random. This process is demonstrated by the loss of the SHG signals at and beyond the ferroelectric phase transition (Curie) temperature. Consequently, flux growth leads to multi-domain^52,96^ KTP crystals. Several reports describe the many efforts expended to manipulate the domain boundaries of KTP^50,51,97^. However, it is possible that H-KTP, synthesized well below (<600 °C) the Currie and melting points, could inherently cultivate single domain crystals and dispose of post-growth alterations.

The as-grown H-KTP crystal was cut into 20 wafers with a thickness of 1 mm along the *z*-plane from beginning to end, as shown in Fig. 10e. The domain of the crystal is characterized by measuring the piezoelectric coefficient *d*_33_. For example, the piezoelectric *d*_33_ value is constant as a single domain; if the sign is opposite, it is an anti-domain region. If the size is smaller than the constant value, it means that the monodomain property is not suitable, and there is an anti-domain in the tested *z-*axis partial region. The test results show that >90% of the areas are single domains, *d*_33_ = 10–10.8 Pc·N^−1^, while *d*_33_ of the F-KTP is about 6 Pc·N^−1^ normally. Next, we tested the *d*_33_ distribution of two crystals grown by the traditional hydrothermal method and MSHT method in the *x*–*y* plane, as shown in Fig. 10c and Fig. 10d, d_33_ has no negative value and the values are similar, indicating that both are a single domain. MSHT method-grown crystal has a smaller *d*_*33*_ variation range, suggesting that it positively affects crystal properties.

### Electric homogeneity

The electrical uniformity discussed here is primarily the dc conductivity *σ*_33_. The *z* polar surface of the two 40 × 15 × 8 mm^3^ plates cuts from H-RKTP single crystals perpendicular to the polar axis for comparison, seen in Fig. 10f. The KTP wafers with polar surfaces are clamped in a specific device for resistance testing, in which the positive electrode uses a metal probe with a diameter of 1 mm as the electrode, the negative electrode uses a conductive rubber plate as the electrode, and the positive and negative electrodes are connected to the resistance measuring instrument to measure a set of data every 5 mm. Our *σ*_33_ measurements are performed at high temperatures, which reduces measurement bias caused by changes in relative humidity. Additionally, the high-temperature *σ*_33_ also provides direct data for applying crystals in high-temperature environments. Indeed, low *σ*_33_ values of 10^−12^–10^−13^ S·cm^−1^ on average at 70 °C have been recorded throughout the H-RKTP sample, and they are relatively uniformly distributed as well. Figure 10h shows the crystal grown by the MSHT method. It can be seen that its *σ*_33_ changes in the range of 40 × 15 mm^2^ in the same order of magnitude, which is different from that of traditional hydrothermal H-RKTP grown by two orders of magnitude (Fig. 10g). Measurement in ultrahigh vacuum (UHV) can also be used to reduce humidity-induced conductivity measurement bias^98^.

## Laser EO modulation based on KTP crystal

**Fig. 11:**
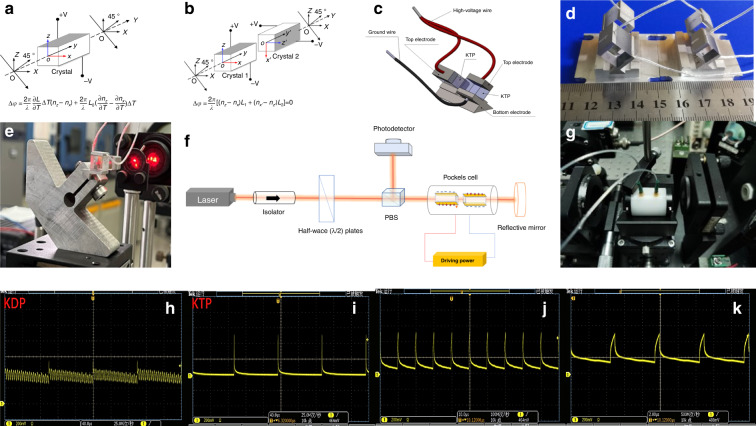
The structure and the piezoelectric ringing of EO Q-switch based on H-RKTP. Schematic of the principal diagram of **a** temperature-controlled single-crystal and **d** temperature-compensated double-crystal EO switches; **b** Photograph and **c** designs of KTP EO Q-switch; **e** Online monitoring device for EO switch pair installation; **f** Experimental setup for characterization of piezoelectric ringing effects (PE); **g** Pockels cell (PC) to be tested; Waveform diagram of the **h** KDP PC under 10 kHz, and KTP PC under **i** 10 kHz, **j** 100 kHz, **k** 200 kHz

One of the primary limitations in applying KTP as an EO device is the intrinsic birefringence of this biaxial crystal. Wang et al. discuss the use of temperature-tuned birefringence compensation of KTP, one H-KTP crystal with a dimension of 4 × 8 × 4 mm^3^ (*x,y,z*) housed in a temperature-controlled oven with 0.1 °C sensitivity^33^. The temperature-controlled KTP PC has a high-polarization extinction ratio, low-dynamic half-wave voltage, and no acoustic ringing at repetition rates up to 30 kHz, but complex and therefore often rarely adopted. Fortunately, to suppress birefringence variations due to temperature changes, another simple and direct approach is to utilize two identical crystals in a compensating mode by Ebbers and Velsko^30^. Two standard concrete methods are to use two crystals of the same length in series, one is to insert a half-wave plate between them (Fig. 11a), and the other is to use the two crystals orthogonally (Fig. 11b, c). The latter is the most used method for most commercial products, while our KTP-based EO switch products also employ this model, as shown in Fig. 11d, e. Specifically, the usual method of compensating for this is to use a pair of crystals, matched in optical thickness, which is then their applied electric fields orientated at 90° relative to each other such that light is polarized along the *x* or *y-*axis say in the first crystal is then polarized along the *z-*axis of the second crystal. The “slow” ray in the first crystal then becomes the “fast” ray in the second and the total static birefringence is thus in theory canceled in the composite crystal pair.

The maximum repetition rate has been determined by the maximum repetition rate of the intracavity EO modulator (PC) used for seeding in and extracting pulses from the amplifier. The PC repetition is limited by the damping rate of the oscillations in the crystal birefringence that arise from the excitation of PER in the crystal. The PER is generated from the piezoelectric effect and can be quantified using the piezoelectric constant related to crystal structure and ferroelectric domains. PER of KDP and KTP were captured at high voltage from 10 kHz to 200 kHz are illustrated in Fig. 11h–k. Even at the highest frequency of 200 kHz, no sign of PER effect was found; on the contrary, the KDP showed an oscillation curve at 10 kHz. Owing to the limitations of the drive power supply, tests at higher frequencies were not performed. However, fortunately, some scholars have used our KTP to obtain lasers with 1.5 MHz repetition frequency^99^. Some scholars have also investigated the PER phenomenon in the modulation frequency range up to 10.8 MHz for KTP in addition to the four fundamental frequencies of the crystal (424 kHz, 766 kHz, 920 kHz, and 1057 kHz), KTP is available for EO modulation of high-repetition frequency lasers below 10.8 MHz^39^.

**Fig. 12:**
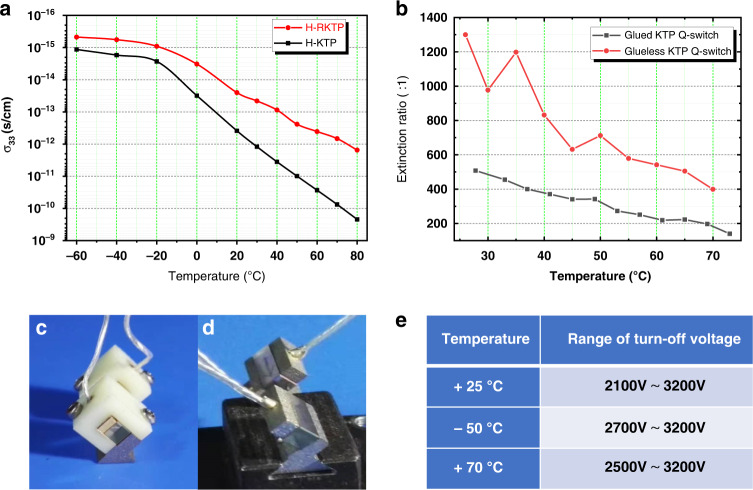
Extinction ratio and thermal effect of the EO modulation based on KTP crystal. **a** Temperature dependence plot of DC conductivity in the range from −60 °C to +80 °C; **b** Variation of the extinction ratio of EO switches with different configurations from room temperature to ~70 °C: glueless (**c**) or glued (**d**); **e** EO switch turn-off voltage range at room temperature, low temperature (−50 °C) and high temperature (70 °C)

Comparison of selected properties of EO crystals, LN, and KTP, respectively. KTP was preferred for EO devices for high-power and high-repetition rate laser because of its lower absorption, superior thermal properties, and similar EO properties. The thermal effect of EO devices is first because KTP is a quasi-1D superionic conductor, so the electrical conductivity is positive depending on temperature, resulting in electron-induced damage and discoloration at high temperatures. Second, accompanying the increase in laser power, one of the primary problems was the thermal defection of the back-propagating beam due to thermally induced refractive index gradients (thermal lensing effect). In addition, although the birefringence change caused by the temperature change can be compensated by the double-crystal structure, the KTP with a large thermo-optic coefficient, and the oscillating laser and pumping light energy absorbed by the front can be compensated and the back two crystals are different, so in the high pumping. Under transport intensity, a thermal depolarization effect will occur, resulting in a poor dynamic extinction ratio of PC, increased insertion loss, and reduced switching performance. On the other hand, when EO switches are used in high-temperature environments such as tropics and deserts, in addition to basic thermal insulation measures, lower conductivity crystals are required. Compared to F-KTP, the *σ*_33_ of H-KTP is relatively low, even lower than most doped modified F-KTP, which can prevent crystal polarization or electro-chromic damage. Nevertheless, to satisfy the use of high-temperature conditions (≥60 °C), this value still needs to be reduced. Therefore, we developed H-RKTP as an EO switch to meet the requirements of a harsh environment. As shown in Fig. 12a, σ_33_ of H-RKTP at 70 °C is 10^−12^ S·cm^−1^, which is lower by 1–2 orders of magnitude than H-KTP. So that it does not cause electrical damage in the range from –60 to 80 °C.

Two crystals of H-RKTP with dimensions 8 × 8 × 11 mm oriented along *y*, *z*, and *x*, respectively, were cut from a boule grown by the MHST method. The half-wave voltage is obtained as 2100–3200 V, and the extinction ratio for s-polarized light is about 1000:1 (30 dB), where the measured optical path diagram is consistent with the measurement of the EO coefficient (Fig. 11f). The extinction ratio of the compensated KTP *Q-*switch with glueless (Fig. 12c) or glued (Fig. 12d) to changes in temperature is demonstrated in Fig. 12b. The extinction ratios of both switches decreased with increasing temperature, compared with the switches without glue having higher extinction ratios in the tested temperature range. The half-wave voltages at high and low temperatures are different, see Fig. 12e, but they do not exceed the range of half-wave voltages at room temperature.

**Fig. 13:**
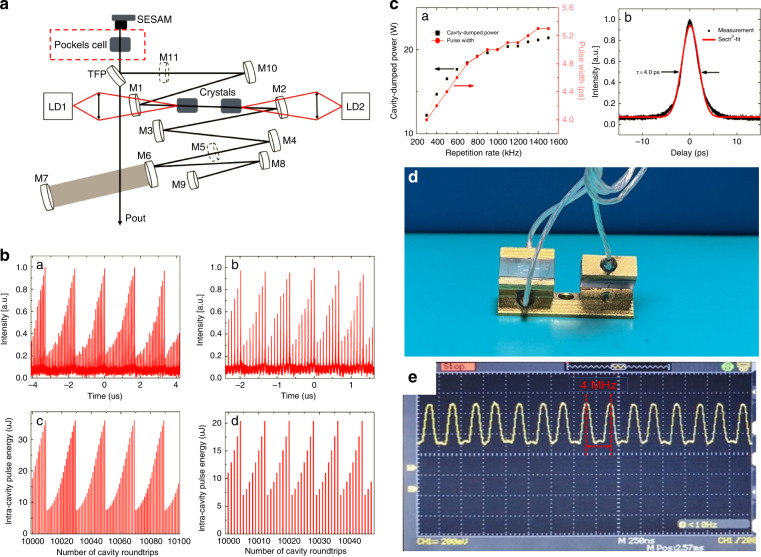
Selected advances in several KTP EO device applications. **a** Schematic of the laser cavity with a KTP Pockels cell (PC); **b** Experimentally formed internal pulse trains with cavity-dumping at repetition rates of: (a) 600 kHz and (b) 1.5 MHz; the corresponding numerical simulations of the intracavity pulse energy at: (c) 600 kHz and (d) 1.5 MHz; **c** Cavity-dumped power and pulse duration (sech^2^-fit) versus dumped repetition rates (left); the measured autocorrelation trace at 300 kHz, assuming a sech^2^-shaped pulse (right). Fig **a**, **b**, and **c** reprinted with permission from ref. ^99^, © 2015 The Optical Society; **d** KTP EO modulator with gold-plated electrode holder for enhanced heat dissipation; **e** Waveform diagram of KTP EO modulator (**d**) at a repetition rate of 4 MHz

High power, high-repetition rate, and few picoseconds Nd: LuVO_4_ oscillator with cavity-dumping were demonstrated by Peng Gao et al.^99^, and shown in Fig. 13a–c. When a KTP PC was inserted in, the oscillator was accomplished with a frequency rate up to 1.5 MHz. During cavity-dumped operation, the laser was fitted with a KTP PC with a repetition rate tuning from 300 kHz to 1.5 MHz (Fig. 13b). The rise and fall times of the PC were both less than 5 ns. The shortest pulse duration is achieved at 4 ps without dispersion compensation. With a cavity-dumping technique, the pulse energy is scaled up to 40.7 μJ at 300 kHz and 14.3 μJ at 1.5 MHz (Fig. 13c).

In a compact fiber CPA laser system, a 500-kHz high-repetition rate PC with two KTP crystals is used to generate pulse trains with controllable pulse number while the pulse energy is kept unchanged, which can satisfy special micro processing need and parallel processing to speed the processing efficiency^100^. KTP PC consists of two paired crystals with the same dimensions of 6 × 6 × 10 mm^3^ used for pulse selection. The high-repetition rate pulse selection is based on KTP PC, which delivers 808 fs, and 105 μJ pulses at a repetition rate of 200 kHz. It can be used as a pulse train generator and a beam splitter for parallel processing. The repetition rate and efficiency can reach as high as 500 kHz and 96%, respectively. Recently, the collaborators tested the high-thermal-conductivity EO modulator based on our H-KTP (Fig. 13d), available to see that the waveform is maintained very well at 4 MHz (Fig. 13e), which will be further applied later.

## Challenges and perspectives of potassium titanyl phosphate crystals

### Larger crystal growth by hydrothermal method

The challenges faced by hydrothermal crystal growth are manifold, and many basic problems are still unsolved, which are inherent in the nature of the hydrothermal method. First, it is very difficult to directly in situ measure internal temperature, concentration, phase, flow, and other parameters for a closed system under extreme conditions. This obstacle to a stable and reliable process can be overcome to some extent by computational fluid simulation. Secondly, high pressure and temperature require the use of high-performance alloys with sufficient strength at elevated temperatures and excellent creep resistance. Furthermore, the dimensioning rules must be strictly followed to minimize stress concentration. Therefore, the enlargement of crystal size is limited by equipment caliber, which is limited by the combination of material size, high-temperature performance, and safety design rules. Unless some new designs of autoclaves are adopted, such as the internal heating type for ammonothermal growing GaN^101^, the inner diameter of the largest autoclave equipment using GH4169 (Inconel 718) or better high-temperature performance alloys in the world has never exceeded our current 120 mm. Now for 120mm × 2.4 m autoclave, crystals of the above size can be grown at one time, reaching 32–40 pieces, with a total weight of about 10Kg. In contrast, the flux method can often only grow one crystal at a time, and the weight of the crystal does not reach near the kilogram level. Like hydrothermally grown quartz^102^, larger-caliber autoclaves for KTP crystal growth are the way forward. The conventional wisdom that hydrothermally grown KTP crystals is limited in size is no longer valid.

### EO modulators replacing RTP for scientific exploration

EO modulators based on RTP were in operation on Mars onboard the Curiosity^103^ and Perseverance^104^. Both integrate the remote Laser Induced Breakdown Spectroscopy (LIBS) for measurements of the elemental composition of samples encountered on Mars. The oscillator of LIBS on Perseverance is Q-switched with an RTP PC to produce about 30 mJ laser at 1064 nm with a pulse duration <5 ns and an *M*^2^ < 2 (−40 to 40 °C). It can identify the chemical and mineral makeup of targets as small as a pencil point up to 7 m distance from the rover^104^. The laser interferometer gravitational wave observatory (LIGO) requires phase modulation of the laser light at RF frequencies. The Enhanced LIGO EOM design uses a crystal of RTP, which has at most 1/10 the absorption coefficient (<500 ppm·cm^−1^) at 1064 nm of the LN crystal from Initial LIGO^105^. At 200 W the RTP should produce a thermal lens of 200 m and higher order mode content of less than 1%, compared to the 3.3 m lens the LN produces at 10 W. The RTP has a minimal risk of damage because it has both twice the damage threshold of LN and is subjected to a beam twice the size of that in Initial LIGO. Benefiting from EOM and a host of other improvements, the Advanced LIGO first direct detected gravitational waves and observed a binary black hole merger on September 14, 2015, at 09:50:45^106^.

Recent EO switch and modulator-based KTP reached a level of maturity that could potentially enable widespread scientific and industrial applications. Conceivably, because of the similarities between RTP and H-KTP, we expect that the H-KTP is also promising to meet the requirements of LIGO and other laser systems. Accordingly, KTP crystals with lower absorption, lower conductivity, larger-sized, and higher thermal stability need to be researched and developed.

### Waveguides in KTP

An optical waveguide can confine the energy of the light in the tiny channel and achieve a high light intensity density, which makes the laser based on waveguide structure have a lower threshold, higher NLO frequency conversion efficiency, and lower EO driving voltage. This makes it the basic component for integrating optics and optoelectronics. The best-known EO material is probably LN with waveguide structure, which has been widely used in telecommunication and renewed attention primarily for its potential application in quantum technologies^2,11,107–109^. There are, nevertheless, limitations associated with LN, in particular for operation at visible wavelengths, as the crystal exhibits a high-optical nonlinearity, excellent mechanical and thermal properties, and high resistance to photorefractive damage. However, KTP suffers from material homogeneities and a large ionic conductivity, which can affect the quality of the QPM grating negatively and result in inhomogeneous waveguide structures, as well as difficulties with reproducibility of fabrication. The waveguides in RKTP are more stable, which is attributed to their lower ionic conductivity and higher spatial homogeneity, allowing repeatable fabrication of homogeneous waveguides and high-quality domain gratings. Nevertheless, the performance of Rb-indiffused channel waveguides in RKTP is still approximately two times lower than the theoretical value, indicating that the ion-exchange parameters can be further improved^110^. In addition to the ion diffusion, other waveguide fabrication techniques for KTP are also highly relevant, such as ion implantation and fs-laser writing. Breakthroughs in these techniques could prove them to be more viable and flexible than the traditional techniques based on ion-exchange. Following a similar path to LN, two-dimensional KTPs with waveguides are also worthy of research and development.

### Periodically poled KTP

Owing to the vigorous development in the field of quantum information, crystals artificially made for quasi-phase-matched (QPM) nonlinear optical spontaneous parametric downconversion (SPDC) received a lot of attention. SPDC is often used with ultrafast lasers to generate photon pairs with precise timing and engineered spectral properties. QPM of periodically polarized KTP (PPKTP) crystals, the brightness of the entangled source was successfully increased from 8.2 × 10^4^ pair·s^−1^·mW^−1^ in 2007^111^ to 10^8^ pair·s^−1^·mW^−1^ in 2018^112^. This greatly improves the quantum communication distance, and in 2017, 1203 km of satellite-to-earth quantum communication was achieved by the “Micius” quantum science experiment satellite^113^.

Bulk crystal with QPM structures interactions is needed for high-power generation. Methods for achieving periodic poling in materials from the KTP is developed. This material combines high nonlinearity with wide transmission range, good power handling capability, and high damage thresholds. Their low coercive field also allows thick crystals to be poled into large aperture QPM devices^114^. On the other hand, the high and varying ionic conductivity in these materials has been identified as important factor complicating the poling process. PPKTP crystals that are either flux or hydrothermally grown have also been tested by Kuzucu et al.^115^. They found that F-KTP exhibited fast degradation in their outputs in the short term and slower decay in the long-term, whereas the hydrothermally grown PPKTP generated steadier outputs. The HKTP with higher performance is looking forward to the breakthrough of commercial periodic polarization technology. At the same time, the combined use of waveguide and periodic polarization technology will open a door for the integrated application of KTP.

### THz-wave technology

Difference frequency generation (DFG) of two lasers in a nonlinear optical material is known to generate coherent radiation in both the low- and high-THz spectra. In recent years, DFG-based THz-wave sources are also been emerging quickly. Compared with LN, KTP has higher laser damage resistance and a larger figure of merit for THz parametric generation. A recent work based on DFG has reported narrow-line far-infrared radiation from KTP off-axis THz parametric oscillator with a maximum output power of about >70 kW at 5.7 THz. With 63% coupling efficiency of the silicon prism atop the KTP crystal, the measured >70 kW far-infrared radiation corresponds to >111 kW powers extracted from the KTP crystal^116^. The radiation source based on KTP has great potential to become a tabletop and economical alternative for the bulky and expensive far-infrared FELs in national facilities. Currently, better cryogenic cooling of the KTP crystal and higher pulse energies, higher repetition rate pump lasers, could also make peak power increases by another 2 orders of magnitude, and frequency increases of 100–1000 times become feasible. Correspondingly, the laser-induced material damage and terahertz absorption of KTP crystals should be paid more attention.

### Second-harmonic generation

In 2024, the French-German MERLIN satellite (Methane Remote Sensing Lidar Mission) will go into Earth orbit to measure concentrations of atmospheric methane with unprecedented precision and thus better understand the sources of this greenhouse gas playing a key role in global warming^117^. For the frequency conversion to the absorption line of methane, an OPO of KTP is implemented in MERLIN. It consists of two KTP crystals in a ring resonator, and the output energy >9 mJ, the wavelength at about 1645 nm in a single pulse, and spectral purity >99.95%. Owing to the large second-order nonlinear optical coefficient and sufficiently large crystal of KTP, its application is difficult to be replaced within the range allowed by the wavelength of KTP. At the same time, if the HKTP with less loss and higher conversion efficiency is used, it will also play a role in improving the laser energy of some wavelengths far lower than the 1064 nm laser.

A new Raman capability in addition to the LIBS mode required a new shorter wavelength produced using a switchable second-harmonic generation (SHG) module in the Perseverance^104^. An SHG conversion efficiency of about 50% is obtained corresponding to about 15 mJ available with a pulse duration of 4 ns. whole qualification temperature range (−40 °C to 40 °C) investigates targets up to 12 m distance from the rover. Compared with the application in the conventional atmosphere, the temperature stability of the application in high-altitude space needs to be paid more attention. The beam is also expanded to 1 cm, illuminating the target with energy >11 mJ, and a 30 KW·mm^−2^ irradiance up to 12 m. Better to provide information at a distance about the mineralogy and molecular structure of the samples under consideration, as well as being able to search for organic compounds that may be associated with past life on Mars.

## Conclusions

Since the excellent EO properties of KTP were realized in 1976 along with the finding of nonlinear properties, many efforts were made to realize its EO applications, based on which we realized the preparation of large size, high damage resistance, and low conductivity crystals further to fabricate it into EO switches and EO modulators. It is possible to grow KTP crystals with a total weight greater than 10 kg at one time (one autoclave per cycle), depending on the development of a large inner diameter autoclave. Through the optimized hydrothermal crystal growth method, the electro-chromic, photo-chromic, and catastrophic laser-induced damages were overcome, and crystals with uniform high optical, electrical, and piezoelectric properties were obtained, making the commercial application of HKTP EO crystals a reality. Recent studies have found that electro-optical devices made of hydrothermal crystals have a high extinction ratio, higher operating temperature, and higher repetition frequency and can be used in complex environments such as high-temperature deserts, extreme cold plateaus, moon, Mars, etc. It can meet the requirements of laser development towards high-repetition frequency, fast switching speed, short pulse width, and tunable wavelength. More importantly, the technological progress of microstructure, such as periodic polarization and optical waveguides, also brings new possibilities for the future of KTP.
